# Urinary ATP as an indicator of infection and inflammation of the urinary tract in patients with lower urinary tract symptoms

**DOI:** 10.1186/s12894-015-0001-1

**Published:** 2015-02-21

**Authors:** Kiren Gill, Harry Horsley, Anthony S Kupelian, Gianluca Baio, Maria De Iorio, Sanchutha Sathiananamoorthy, Rajvinder Khasriya, Jennifer L Rohn, Scott S Wildman, James Malone-Lee

**Affiliations:** Division of Medicine, University College London, Archway Campus, London, UK; Department of Statistics, University College London, London, UK; Medway School of Pharmacy, The Universities of Kent and Greenwich at Medway, Chatham, Kent, UK; Research Department of Clinical Medicine, Division of Medicine, University College London, Wolfson House, 2 – 10 Stephenson Way, NW1 2HE London, UK

**Keywords:** Lower urinary tract symptoms (LUTS), Adenosine-5′-triphosphate (ATP), Urinary tract infection (UTI)

## Abstract

**Background:**

Adenosine-5′-triphosphate (ATP) is a neurotransmitter and inflammatory cytokine implicated in the pathophysiology of lower urinary tract disease. ATP additionally reflects microbial biomass thus has potential as a surrogate marker of urinary tract infection (UTI). The optimum clinical sampling method for ATP urinalysis has not been established. We tested the potential of urinary ATP in the assessment of lower urinary tract symptoms, infection and inflammation, and validated sampling methods for clinical practice.

**Methods:**

A prospective, blinded, cross-sectional observational study of adult patients presenting with lower urinary tract symptoms (LUTS) and asymptomatic controls, was conducted between October 2009 and October 2012. Urinary ATP was assayed by a luciferin-luciferase method, pyuria counted by microscopy of fresh unspun urine and symptoms assessed using validated questionnaires. The sample collection, storage and processing methods were also validated.

**Results:**

75 controls and 340 patients with LUTS were grouped as without pyuria (n = 100), pyuria 1-9 wbc μl^-1^ (n = 120) and pyuria ≥10 wbc μl^-1^ (n = 120). Urinary ATP was higher in association with female gender, voiding symptoms, pyuria greater than 10 wbc μl^-1^ and negative MSU culture. ROC curve analysis showed no evidence of diagnostic test potential. The urinary ATP signal decayed with storage at 23°C but was prevented by immediate freezing at ≤ -20°C, without boric acid preservative and without the need to centrifuge urine prior to freezing.

**Conclusions:**

Urinary ATP may have a role as a research tool but is unconvincing as a surrogate, clinical diagnostic marker.

## Background

“Lower Urinary Tract Symptoms” (LUTS) is a collective term describing [1] urinary storage problems such as frequency, urgency and urge incontinence; [2] voiding difficulties such as hesitancy, reduced stream, intermittency and incomplete voiding; [3] sensory symptoms that include various experiences of pain; and [4] stress urinary incontinence. There is considerable overlap between these symptoms [1] so that diagnostic categorisation is difficult. Whatever the symptom mix, the exclusion of urinary tract infection (UTI) is a mandatory first step in the assessment of all LUTS [2]. Whilst acute UTI is not diagnostically challenging, in the case of LUTS without acute frequency and dysuria, exclusion of infection poses a diagnostic challenge.

There are good reasons to scrutinise urinary adenosine-5′-triphosphate (ATP) as a possible surrogate marker of UTI since the reliability of popular diagnostic methods used to exclude UTI have been questioned [3-6]. Published guidelines across Europe, USA and the UK reveal significant discrepancies in the choice of a quantitative threshold used to define significant bacteriuria. The clean-catch, midstream urine (MSU) sample culture in the UK and Europe commonly uses the Kass (1957) [7] criterion of 10^5^ colony forming units (cfu) ml^-1^ of a single species of a known urinary pathogen. Kass drew these data from 74 women with acute pyelonephritis and 337 normal controls, a select sample unrepresentative of wider lower urinary tract symptoms (LUTS). Despite its limitations, this criterion has become a ubiquitous reference standard and has been challenged by several groups [6,8]. The European Associate of Urology (EUA) guidelines for urological infections emphasise that no single threshold can be applied in all clinical situations. The urinary dipstick tests for nitrite and leucocyte esterase are also commonly used as a bedside screening test for infection and as a measure of a positive urine culture. However the use of urinary dipstick have been validated against the urine culture to a threshold of 10^5^ colony forming units (cfu) ml^-1^ and given the recent criticism of the Kass criterion, urinary dipstick have also recently been found to be unreliable [3,4,9].

Urinary tract ATP has attracted intense interest in the last 30 years for its pharmacological and pathophysiological associations. There are great hopes that purinergic receptor manipulation might influence detrusor motor function and urothelial afferents [10], achieving therapeutic benefit. ATP is an important urothelial cell distress signal [11] and is released by inflammatory cells and bacteria [12]. Urinary tract infection featuring bacterial invasion, urothelial distress and an innate immune response involving recruitment of inflammatory cells, should be associated with increased urinary ATP levels. Indeed, high levels of ATP have been detected in the urine of patients with interstitial cystitis and acute UTI with a positive urine culture [13]. Increased ATP has also been shown to be released from cultured urothelial cells infected with uropathogenic *E.coli* (UPEC) [14], and UPEC also produce ATP when cultured *in vitro* [15]. It has been postulated that ATP may reflect microbial biomass and hence ATP increases as the amount of bacteria present increases. Currently ATP levels are used widely in the food, water and sanitation industry as a measure of bacterial contamination [16].

Given the problems with current tests [3], developing alternative diagnostic assays is a high priority. We therefore sought to scrutinise the performance of urinary ATP to test for potential as a surrogate measure of inflammation and infection when assessing patients with chronic LUTS. The experiment was divided into two parts; [1] a clinical experiment that evaluated urinary ATP in patients with LUTS and controls, comparing urinary ATP with symptoms, microscopic pyuria and urine culture results; and [2] a laboratory experimental series that explored the factors that could influence sample collection, storage and preservation. As urine contains native ATPase activity, the time-decay curve of urinary ATP from collection to processing was evaluated. Boric acid crystals, which are commonly used as a urinary preservative, have been shown to prevent microbial swarming [17] and boric acid has a preservative influence on white cells [18]. We therefore studied the effects of the use of urinary preservative boric acid, storage temperature and the effect of centrifugation on urinary ATP concentration.

## Methods

### Ethical approval

Ethical committee approval for this study, including all study documentation, was obtained from the Whittington and Moorefields Research Ethics Committee. All study participants gave informed written consent to participate in the study and the process was documented as per Good Clinical Practice (GCP) and MHRA guidelines. The participants were assigned randomly generated study numbers which were used to anonymise all data and samples, and analysis was carried out by blinded researchers.

### Patients and symptom collection

Adult patients presenting with lower urinary tract symptoms were recruited from incontinence clinics from October 2009 to October 2012 and informed consent obtained. We compared urine samples from 75 healthy controls and 340 patients presenting with LUTS. The demographic data can be seen in Table 1. The control group consisted of 49 females and 26 males, with mean age 38.2 yrs (95% CI 34.5 - 41.8). Within the LUTS group there were 314 females and 26 males, with a mean age of 58.6 yrs (95% CI 56.8 - 60.4). All patients completed detailed validated LUTS questionnaires covering 38 symptoms, including frequency, nocturia, urgency, incontinence episodes, symptoms relating to storage function, voiding problems, stress urinary incontinence and pain, which were recorded on a bespoke clinical database. Control subjects completed questionnaires thereby confirming absence of symptoms. There are popular, validated symptom scores such as the ICIQ series [19] which are suitable as intervention outcome measures because changes in individual scores can be normalised for group comparisons. However, adjectival scaling such as ‘bother’, when deployed in cross-sectional, descriptive work, is vulnerable to semantic interpretation differences, and considerable error may occur [20]. To avoid this, we used validated scales that measure symptoms dichotomously and achieve scaling by counting the contexts in which symptoms occur. These are effective measures for cross-sectional observation studies [21-23].

**Table 1:** **Demographic data**

	**Controls**				**LUTS patients**			
Gender male	N = 26				N = 26			
Gender female	N = 49				N = 314			
No pyuria	N = 58 (female = 35, male = 23)				N = 100 (female = 92, male = 8)			
Any pyuria	N = 17 (female = 14, male = 3)				N = 240 (female = 222, male = 18)			
	**Mean**		**Std deviation (sd)**		**Mean**		**Std deviation (sd)**	
Age (years)	38.2		15.8		58.6		16.6	
	**Mean**	**Median**	**sd**	**Quartile range**	**Mean**	**Median**	**sd**	**Quartile range**
24 hour frequency	6.2	6.2	6.0 to 7.0	6.0 to 7.0	9.2	8.0	8.0 to 10.0	6.0 to 11.0
24 hour incontinence	0	0	0.0 to 0.0	0.0 to 0.0	0.8	0.5	0.5 to 1.2	0.0 to 1.0
Number of urgency symptoms	0	0	0.0 to 0.0	0.0 to 0.0	2.8	2.0	2.0 to 3.6	0.0 to 4.0
Number of pain symptoms	0	0	0.0 to 0.0	0.0 to 0.0	0.4	0.0	0.2 to 0.6	0.0 to 0.0
Number of stress inc symptoms	0	0	0.0 to 0.0	0.0 to 0.0	0.3	0.0	0.08 to 0.5	0.0 to 0.0
Number of voiding symptoms	0	0	0.0 to 0.0	0.0 to 0.0	1.4	0.0	1.0 to 1.8	0.0 to 2.0
Number of LUTS	0	0	0.0 to 0.0	0.0 to 0.0	5.0	3.5	4.0 to 6.0	1.0 to 6.0

### Midstream urine (MSU) collection

Samples were obtained by the midstream clean-catch method. Patients were given detailed instruction on how to collect a meticulous midstream urine sample and avoid perineal contamination. This included use of an antiseptic wipe to clean the genital area prior to voiding and use of a sterile large flexible container introduced to collect the urine mid-flow and removed before completion of voiding.

### Routine urine culture

The urine was cultured using the Kass [7] threshold for significance which is the standard method of analysis in UK-NHS practice. A 1 μl loop of urine was plated on a chromogenic agar plate and incubated at 37°C for 24 hours. Cultures were reported as positive if there was bacterial growth of a single urinary pathogen of greater than 10^5^ cfu ml^-1^, negative bacterial growth if less than 10^5^ cfu ml^-1^ and reported as mixed growth if there was more than one uropathogen with total growth greater than 10^5^ cfu ml^-1^. Although 10^5^ cfu ml^-1^ threshold is known to be inadequate [6,3,24], we included it in this study because it such a ubiquitous gold standard.

### Microscopic leucocyte count

A fresh aliquot of urine was examined by microscopy. 10 μl of urine was loaded into a Neubauer haemocytometer counting chamber [25] and examined by light microscopy (magnification x200) for leucocytes.

### Blinding

Microscopy and ATP analysis were performed by researchers blinded to the details or symptoms of the participants. Samples presented for analysis were identified only by a randomly generated four-digit study number.

### ATP analysis

Samples were processed using Sigma-Aldrich Adenosine 5′-triphosphate (ATP) Bioluminescent Assay Kit, at an approximate cost of £250, which is able to detect concentrations of 2 × 10^-12^ to 8 × 10^-5^ mol/L. Urine samples stored at -80°C and -20°C were thawed in a water bath in room temperature (23°C) and processed according to the manufacturer’s instructions. Reagents were prepared as per the manufacturer’s recommendation with measures included to prevent degradation. The standard assay kit (SigmaAldrich; Missouri, USA) applied a bioluminescent reaction involving the breakdown of luciferin by luciferase, which requires ATP. The average luminescence recorded, in relative light units (RLU), was proportional to the concentration of ATP. This was converted to ATP moles ml^-1^ using a standard curve.

### Evaluation of the effect of time on urinary ATP

Urine samples used to assess stability of ATP over time were stored at room temperature (23°C). Aliquots were taken at 0 hours (immediately), 12 hours, 24 hours, 48 hours, 168 hours and frozen at -80°C.

### Evaluation of the effect of storage temperature on urinary ATP

The literature tends to recommend storage at -80°C; however, this is not convenient for many clinical services. The effects of freezing at -80°C and -20°C were therefore studied. 2 ml aliquots of fresh urine were taken from each participant. One aliquot was frozen immediately after microscopy and stored at -80°C and another at -20°C.

### Evaluation of the use of boric acid preservative on urinary ATP

We studied the effects of boric acid preservation on decay of urinary ATP. 10mls of urine was introduced into pre-prepared boric acid tubes (Becton Dickinson Vacutainer® C & S Preservative Urine Tubes for Culture and Sensitivity) and stored at room temperature. Aliquots were taken at 0 hours (immediately), 12 hours, 24 hours, 48 hours, 168 hours and frozen at -80°C.

### Evaluation of the effect of centrifugation on urinary ATP

Urinary ATP may originate from several sources including bacteria, urothelial cells and white cells. Therefore, we sought to discover whether centrifuging the urine alters the assay result, either by removing cells from the supernatant, or by lysing cells in the process [26]. An aliquot of urine was spun at 620 g for 5 minutes and the supernatant was frozen at -80°C.

All of the urine aliquots for the clinical experiments were frozen immediately at -80°C. These samples were processed for urinary ATP between eight to twelve weeks after collection and storage. The frozen urine aliquots were thawed to room temperature (23°C) using a water bath and then immediately analysed using the standard luciferin-luciferase assay and protocol, which was described earlier.

### Statistics

We used multivariate linear regression analyses to scrutinise the log_10_ ATP as the response variable using two models. In the first, the explanatory variables were gender (0 = female, 1 = male); age; average 24-hour frequency; average 24-hour incontinence; number of stress incontinence symptoms, pain symptoms, voiding symptoms and OAB symptoms; the presence or absence of any pyuria (0 = none, 1 = any pyuria); and the MSU culture result (0 = negative, 1 = positive). In the second model we looked more closely at the effect of the degree of pyuria. Pyuria was grouped as zero pyuria, pyuria 1-9 or pyuria ≥10, subgroups which are currently used by most clinicians, and these were referenced to control samples. The sample had 83% power to detect a .04 increment in R^2^_,_ if ten predictor variables were included in the regression model with alpha = 0.05. In the laboratory experimental series, paired data was collected and hence we used different statistical analysis methods to the clinical experiment. We used the paired t-test to analyse the difference in log_10_ ATP between paired samples stored at -20°C and -80°C; paired samples stored with and without boric acid; and paired samples centrifuged or uncentrifuged. The diagnostic potential of urinary log_10_ ATP was assessed by ROC plots using Positive MSU at 10^5^ cfu ml^-1^ of a pure isolate of a known urinary pathogen; pyuria ≥ 10 wbc μl^-1^ and pyuria > 0 wbc μl^-1^.

## Results

**Figure 1:**
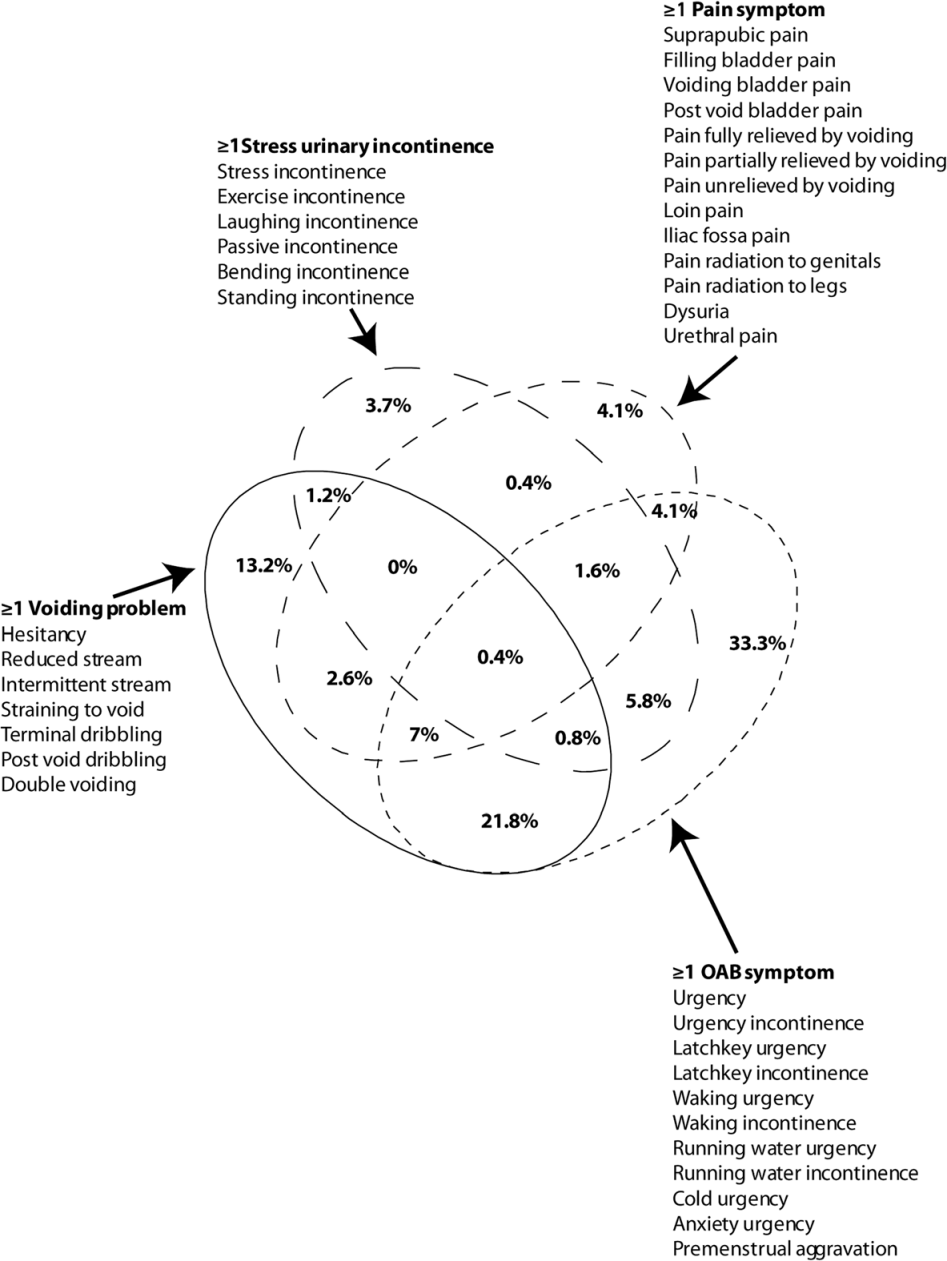
**Venn diagram of symptom analysis.** A four-way Venn diagram illustrating the overlap of symptom amongst the patients studied. The ellipses circumscribe patients who had one or more symptoms in the particular subset. The diagram is not scaled to the size of sets.

We compared urine samples from 75 healthy controls and 340 patients presenting with LUTS. The demographic data can be seen in Table 1. The patients cohort was grouped in the first model; with pyuria (≥1 wbc μl^-1^) or without pyuria (0 wbc μl^-1^). For the second model we used categorical scaling so that we compared pyuria 1-9 wbc μl^-1^ (n = 120) and pyuria ≥10 wbc μl^-1^ (n = 120) with a baseline factor of zero pyuria (0 wbc μl^-1^). Of those with LUTS, 33.3% had only OAB symptoms, 4.1% had pain alone, 3.7% had only stress incontinence and 13.2% had only voiding dysfunction. Patients had a median of 3.5 LUTS (quartile range 1 to 6). The overlap of symptoms is illustrated in Figure 1.

**Figure 2:**
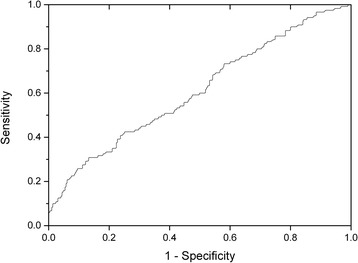
**Receiver-operator characteristics (ROC) curve for urinary ATP for the diagnosis of UTI.** The ROC analysis shows an area under the curve of 0.6 for pyuria ≥ 10 wbc μl^-1^. These data imply that there is no useful diagnostic role for this assay.

Log_10_ transformation of ATP changed the skewness from 4.2 to -0.3 and kurtosis from 27.5 to 1.1. The results of the two regression analyses are shown in Table 2. It can be seen that female gender was associated with higher predictions of the log_10_ ATP. However given the small number of males and predominance of female patients, which is a reflection of this condition, limited discriminatory power precludes extrapolation. Voiding symptoms were also predictive of higher log_10_ ATP. Interestingly, voiding symptoms in both sexes have been reported to be associated with inflammatory disease of the lower urinary tract [22]. A positive culture result predicted lower log_10_ ATP. The first regression model shows that the presence of any pyuria was not predictive of higher log_10_ ATP. The second regression model demonstrates that a pyuria ≥10 wbc μl^-1^ was predictive of a higher log_10_ ATP; however, lower levels of pyuria 1-9 wbc μl^-1^ were not. These data do demonstrate that urinary ATP is elevated in association with inflammation but that urinary ATP lacks the discriminating properties at lower levels of pyuria, which are necessary for a useful clinical surrogate marker. This is illustrated by the ROC analysis which showed an area under the curve of 0.6 for a positive culture; 0.5 for pyuria > 0 wbc μl^-1^ and 0.6 for pyuria ≥ 10 wbc μl^-1^ (Figure 2). These data imply that there is no useful diagnostic role for this assay. The regression analyses showed that, age, average 24-hour frequency, average 24-hour incontinence, the number of urgency, stress incontinence, and pain symptoms provided no substantial explanation of the variance of urinary log_10_ ATP.

**Table 2:** **Output from regression analysis**

**Model 1 pyuria described by dichotomy**				
			**95% confidence interval for B**	
	**B coefficient**	**p**	**Lower bound**	**Upper bound**
(Constant)	-8.026	.000	-8.400	-7.652
Age	.004	.197	-.002	.009
**Gender 0 = female, 1 = male**	**-.537**	**.001**	**-.865**	**-.209**
**MSU 0 = negative 1 = positive**	**-.346**	**.004**	**-.582**	**-.110**
Average 24-hour frequency	-.004	.743	-.026	.019
Average 24-hour incontinence	-.001	.990	-.092	.090
Number of stress incontinence symptoms	-.065	.179	-.161	.030
**Number of voiding symptoms**	**.124**	**.000**	**.061**	**.187**
Number of pain symptoms	.082	.382	-.103	.267
Number of urgency symptoms	-.030	.163	-.072	.012
Pyuria 0 = none 1 = any	.157	.107	-.034	.349
**Model 2 pyuria described by ordinal scale**				
			**95% confidence interval for B**	
	**B coefficient**	**p**	**Lower bound**	**Upper bound**
(Constant)	-8.058	.000	-8.431	-7.685
Age	.002	.404	-.003	.008
**Gender 0 = female, 1 = male**	**-.522**	**.002**	**-.845**	**-.199**
**MSU 0 = negative 1 = positive**	**-.406**	**.001**	**-.642**	**-.171**
Average 24-hour frequency	-.002	.878	-.024	.021
Average 24-hour incontinence	-.024	.607	-.114	.067
Number of stress incontinence symptoms	-.060	.210	-.154	.034
**Number of voiding symptoms**	**.106**	**.001**	**.043**	**.169**
Number of pain symptoms	.085	.356	-.096	.266
Number of urgency symptoms	-.024	.257	-.066	.018
Pyuria1 to 9 wbc μl^-1^	.207	.059	-.008	.422
**Pyuria ≥ 10 wbc μl** ^**-1**^	**.432**	**.000**	**.203**	**.662**
Dependent variable is urinary log_10_ ATP concentration

### Urinary ATP decay over time

**Figure 3:**
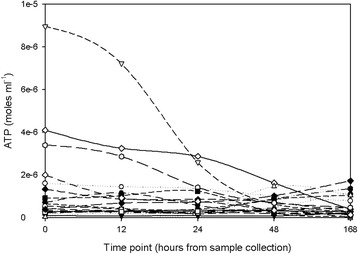
**The ATP decay curves for a subset of 20 urine samples.** The rate of decay is substrate concentration dependent; such that a sample with a higher ATP concentration shows a more marked decay. It is therefore important to assay or freeze immediately after collection of the urine specimen to preserve the ATP signal.

A subgroup of 20, randomly selected patient samples was used to plot the urinary ATP concentration in samples at differing time points after collection; 0 hours, 12 hours, 24 hours, 48 hours, 168 hours. Aliquots were taken at each point and frozen at -80°C and stored for assays in batches. The ATP concentration fell with time with the rate of decline very dependent on the initial concentration, as illustrated in Figure 3, where the time course of each sample is plotted.

### Urinary ATP and effect of storage with boric acid preservative

**Figure 4:**
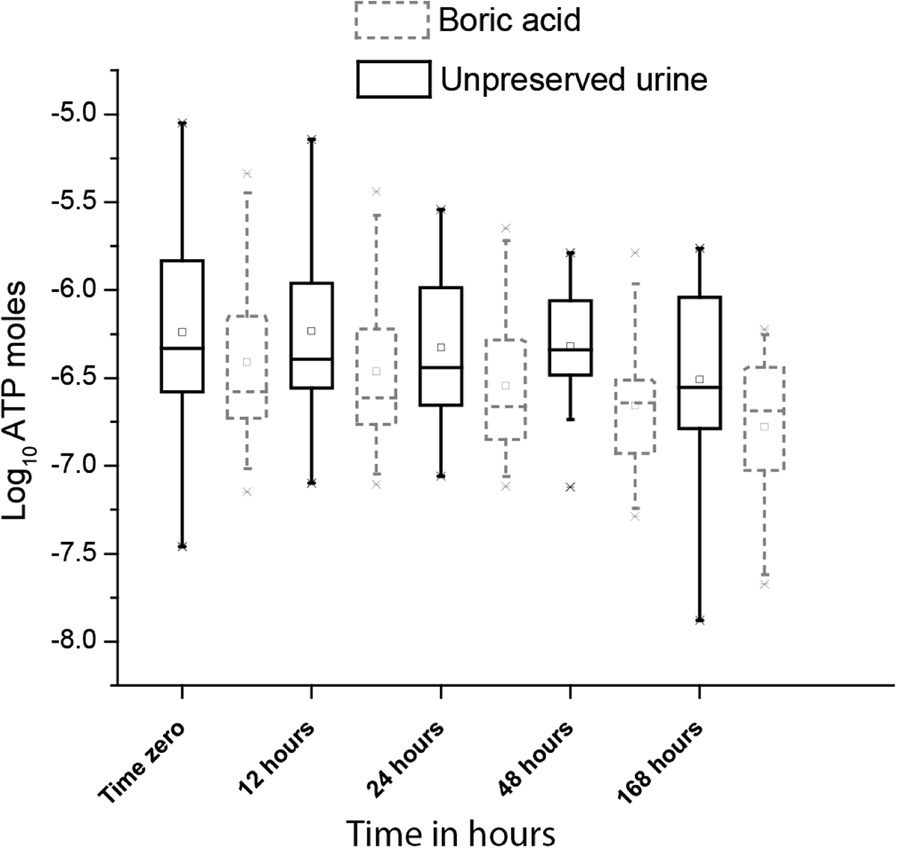
**A box plot showing the effects of boric acid preservative on urinary ATP decay.** The box plot shows a subset of 20 paired samples to test the effect of boric acid as a urinary preservative on the decay of urinary ATP. Urinary ATP was measured at 12 hour intervals in urine samples with and without boric acid.

Figure 4 shows box plots of the log_10_ ATP concentration at each time point comparing the effect of boric acid. An analysis of 20 paired samples at 24-hours demonstrated the significance of this difference: mean log_10_ ATP (moles) in samples stored without boric acid was -6.3 log_10_ moles and in samples stored at with boric acid was -6.5 log_10_ moles (95% CI difference 0.15 to 0.29, t = 6.2, p < .001). (*Put significance at end of paragraph as you did for the following paragraphs)* These data show that boric acid caused loss of ATP.

### Urinary ATP and effect of storage temperature

Comparison of 30 paired samples of urine stored at -20°C and -80°C showed no significant difference in ATP concentration: mean log_10_ ATP (moles) in samples stored at -20°C was -6.7 log_10_ moles and in samples stored at -80°C was -6.8 log_10_ moles (95% CI difference -0.09 to +0.01 , t = -1.7, p = .1). Thus storage at -20°C for 8 weeks would seem reasonable.

### Urinary ATP and effect of centrifugation

Comparison of 30 paired samples of urine unspun and spun showed that the supernatant urine had a slightly lower level of ATP: mean log_10_ ATP (moles ml^-1^) in uncentrifuged samples = -6.9 log_10_ moles and in the supernatant after centrifuge mean = -6.8 log_10_ moles (95% CI difference 0.03 to 0.1, t = 3.5, p = .002). Whilst statistically different, this is a very small difference and of little clinical significance.

## Discussion

ATP has been proposed as a potential clinical marker of infection for both acute and chronic LUTS [10,27]. To avoid premature use, when assessing the diagnostic potential of test, it is important to assess first whether the measure explains the symptoms and other manifestations of the disease of interest [28]. The data published here demonstrate that urinary ATP does not offer any additional benefit to tests currently used in screening for infection in patients presenting with LUTS, and therefore does not show promise for future development of a diagnostic test for this particular group. This observation is confirmed in the ROC curves that explore sensitivity and specificity properties.

Our results demonstrate that patients with LUTS and any pyuria do not manifest a significantly raised urine ATP concentration. It is only when there is pyuria of greater than 10 wbc μl^-1^, which is a currently used marker of urinary infection, that there is a significantly raised urinary ATP. However, this signal was not a discriminating marker for low levels of lower urinary tract inflammation (pyuria 1-9 wbc μl^-1^) as shown by our second regression model, and it is in these clinical circumstances that there is greatest need for novel clinical surrogate markers. It has been shown that low-level pyuria [26], voiding symptoms [22], overactive bladder symptoms [24] and pain symptoms [23] all correlate with urinary infection. Only voiding symptoms explained a small amount of the variance in ATP. These findings are therefore discouraging.

Counter-intuitively, urinary ATP was lower, given a positive urine culture. This unexpected result may reflect the fact that there were so few positive cultures (16.9%) that there weren’t enough data to draw meaningful conclusions. Increased microbial dephosphorylation of ATP to adenosine, which we did not assay, may also be a contributing factor. In addition, the urinary ATP reflects the urinary microbial biomass directly post void and there may be significant variation in the urinary biomass at the time of culture secondary to transportation and processing delay.

There was an unbalanced sample size with fewer control participants with a lower average age, and this reflects the common difficulty of finding older subjects without any urinary tract symptoms. The controls in this experiment were all asymptomatic. We did control for these differences in the analysis but would nevertheless wish to avoid wide inferential generalisations. The essence of this study was to scrutinise the discriminating properties amongst patients. The recruitment field was also predominantly female which may explain the gender difference. The small numbers of males were nevertheless included in the analysis as the model was sufficiently powered for this variable, which required ≥20 for each independent variable. The sample was powerful enough to detect the influence of voiding symptoms, pyuria and culture, despite the wide variance. Urinary ATP concentrations proved independent of age and the number and nature of LUTS symptoms, other than voiding symptoms. The association with voiding symptoms was not surprising because in patients with chronic LUTS, these have been reported to be correlated with pyuria in both genders, in clear contrast to OAB and pain symptoms [22].

We found that urinary ATP could not pick out low levels of inflammation (pyuria 1-9 wbc μl^-1^) when used alone, a situation where clinicians need most help. Current dipstick methods and direct urinary microscopy are able to identify pyuria when it is abundant [26], so urinary ATP would offer no additional advantage. There can be little doubt that ATP plays a very significant role in the pathophysiology of urinary tract disease [29,30] but it would seem that this does not translate into a useful role as a clinical test when used alone.

The laboratory experimental series showed that urine samples should be processed immediately or frozen and stored at below -20°C. We demonstrated significant urinary ATP decay with time when samples were left at room temperature, with the rate being substrate dependent. We also found that storage of urine at -20°C is adequate and this therefore allows for wider use of standard freezers as opposed to the specialised -80°C devices. The centrifuge studies examined the effect of the removing cellular material. The supernatant showed marginally lower levels of ATP. This could be attributed to increased ATPase activity from burst cells or it may have resulted from delayed freezing, allowing additional time for enzyme activity. Additionally, centrifugation results in the removal of biomass and hence this may explain the lower levels of urinary ATP. This marginal difference, though statistically significant, may not be clinically significant; nevertheless, centrifuging of urine prior to freezing or analysis is an additional labour worth avoiding. Boric acid proved counterproductive and so should not be used as a preservative.

## Conclusion

In summary these data discourage the idea that urinary ATP should be developed as a clinical surrogate test for UTI in patients presenting with LUTS. This assay does not appear more effective than markers used in current clinical practice [3,26]. However, abundant urinary ATP is certainly evident amongst patients with lower urinary tract symptoms and dysfunction and these data do encourage continued interest in the pharmacology and pathophysiology of purinergic functions in the bladder. For those committed to these avenues of discovery, we provide valuable data on human sample processing, storage and assay.

In conclusion, urinary ATP does not improve on the use of microscopy as a surrogate marker of urinary tract infection in patients presenting with LUTS and is therefore not a promising clinical diagnostic marker. We need to continue to explore other potential markers that can be used to screen LUTS patients for UTI, particularly applicable to those that have lower levels of pyuria, where significant disease may currently be overlooked [26]. The relevance of measuring ATP to study the pathophysiology of the lower urinary tract is still very evident and in that context urine is a useful biological sample.

## Abbreviations

ATP: Adenosine-5'-triphosphate
CFU: Colony forming units
LUTS: Lower urinary tract symptoms
MSU: Midstream urine
UPEC: Uropathogenic E. coli
UTI: Urinary tract infection
